# Extracellular Vesicles in Chronic Demyelinating Diseases: Prospects in Treatment and Diagnosis of Autoimmune Neurological Disorders

**DOI:** 10.3390/life12111943

**Published:** 2022-11-21

**Authors:** Leyla A. Ovchinnikova, Arthur O. Zalevsky, Yakov A. Lomakin

**Affiliations:** Shemyakin-Ovchinnikov Institute of Bioorganic Chemistry, RAS, 117997 Moscow, Russia

**Keywords:** extracellular vesicle, exosomes, microvesicles, EVs, EAE, biomarker, multiple sclerosis, autoimmunity, CNS, miRNA

## Abstract

Extracellular vesicles (EVs) represent membrane-enclosed structures that are likely to be secreted by all living cell types in the animal organism, including cells of peripheral (PNS) and central nervous systems (CNS). The ability to cross the blood-brain barrier (BBB) provides the possibility not only for various EV-loaded molecules to be delivered to the brain tissues but also for the CNS-to-periphery transmission of these molecules. Since neural EVs transfer proteins and RNAs are both responsible for functional intercellular communication and involved in the pathogenesis of neurodegenerative diseases, they represent attractive diagnostic and therapeutic targets. Here, we discuss EVs’ role in maintaining the living organisms’ function and describe deviations in EVs’ structure and malfunctioning during various neurodegenerative diseases.

## 1. Introduction

Extracellular vesicles (EVs) are remarkable omnipresent particles responsible for intercellular communication in various living organisms. The interest in this field is rapidly growing, according to the mounting body of EV-associated publications. EVs contribute to almost every possible metabolic reaction in our body. The major characteristics of EVs, such as micro- or nano-scale size, high biocompatibility, and the ability to cross the blood-brain barrier (BBB), make them ideal therapeutic tools. Naturally occurring EVs transfer nearly all cellular components: they contain amino acids, proteins, including various transcriptional factors, DNA fragments, various RNAs (siRNAs, mRNAs, miRNAs, non-coding RNAs) or even carbohydrates and lipids incorporated into their membrane [1]. EVs are secreted by all cell types and are present in all body fluids. Their composition and quantity may change with the development of various diseases, implicating their role in the pathology process [2]. Thus, uncovering these alterations has a huge diagnostic potential.

**Figure 1:**
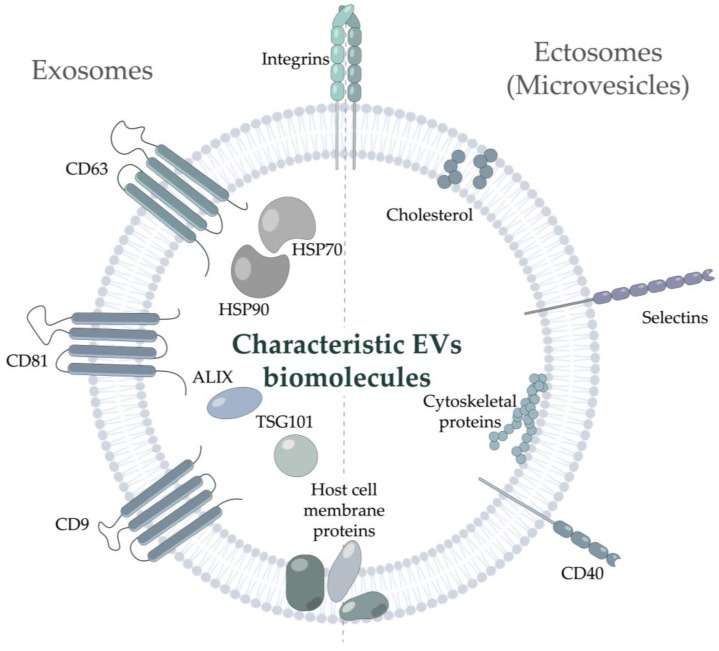
Markers and characteristic molecules of Extracellular vesicles. Typical vesicular markers of exosomes and ectosomes (microvesicles) are shown. ALIX—ALG-2-interacting protein X; HSP—Heat Shock Protein; TSG101—Tumor Susceptibility Gene 101 protein.

EVs are a highly heterogeneous group of bilayer nanoparticles of various origins, incapable of replication [3]. According to MISEV2018 (The first Minimal Information for Studies of Extracellular Vesicles) guide of the International Society for Extracellular Vesicles, EVs should be described based on their (i) physical characteristics, such as size (“small EVs”, “medium/large EVs”) or density (low, middle, high), (ii) biochemical composition, or (iii) description of either conditions or the cell of origin [3]. Nonetheless, the historically accepted classification based on EVs’ biogenesis is generally applied. According to this classification, EVs are divided into two major classes, exosomes and ectosomes [4]. The diameter of the smallest vesicles, exosomes, varies from 30–50 nm to 100–150 nm. They originate from the multivesicular bodies and pass through complex endosomal machinery during secretion. The exosome markers include tetraspanins (CD9, CD63, CD81) [5], the endosomal protein tumor susceptibility gene 101 (TSG101), 70-kDa heat shock protein 70 (HSP70) [6], and ALG-2-interacting protein X (ALIX) [7]. Notably, accumulating studies have demonstrated that integrins can be identified in exosomes not only under pathophysiological conditions [8] but should be considered commonly identified exosome-related proteins [9]. Another class of vesicles with a diameter in the range of 50–1000 nm, ectosomes or microvesicles, is released by the cell self-membrane budding [10,11]; it is rarely characterized by any specific markers present. Several studies noted the presence of CD40 [12], selectins, tetraspanins, and integrins [13] in ectosomes. Their membrane may also contain some host cell proteins and lipids [14,15] (Figure 1). However, this classification is likely to be further revised due to new classes of EVs-like nanoparticles still emerging. For example, exomeres, recently discovered non-membranous nanoparticles (<50 nm), have not been described in detail yet [16]. Moreover, ultracentrifugation of the supernatant of exomeres revealed novel nanoparticles, termed supermeres [17]. However, there is no consensus regarding whether they can be considered true EVs or not. Of note, a distinct class comprises the supramolecular complexes produced by T cells during the immunological synapse development: polarized exosomes [18] and synaptic ectosomes [19], EVs formed in a trans-synaptic vesicle [20], as well as supramolecular attack particles (SMAPs), lacking the phospholipid membrane and assembled from the thrombospondin-1 shell [21].

In the nervous system, EVs are essential in signal transmission both in the central nervous system (CNS) and in the peripheral nervous system (PNS). Vesicles produced by astrocytes, neurons, oligodendrocytes, microglia, and Schwann cells have been already described in substantial detail [22,23,24]. According to the classification, based on biochemical composition, EVs are distinguished from synaptic vesicles (SVs), released at chemical synapses and predominantly containing the classical neurotransmitters. Apart from their role in a normal physiological response such as non-synaptic neural and vascular-neural communication through BBB, EVs produced by neural cells provide unique information regarding the state of the nervous system in general. As EV cargoes, including proteins or RNAs, may differ in healthy and pathological states [25], their content might serve as a predictor of pathological conditions and neuroinflammation.

Recent studies have obtained some important pieces of the puzzle on how EVs function during autoimmune states, their nature, and biogenesis. Here, we overview the prospects of harnessing EVs as novel therapeutic targets and potential biomarkers of autoimmune neurodegenerative diseases. We also highlight the advantages and limitations of using EVs as next-generation molecular tools.

## 2. The Role of EVs in the Nervous System

Both CNS and PNS comprise a vast number of neuronal and neuroglial cell types. The homeostasis of the nervous system significantly relies on intercellular communications [26]. In the nervous system, cells communicate via gap junctions, cell adhesion, and EVs loaded with neurotransmitters and growth factors. EVs exhibit the potential to regulate cell proliferation in developing neural cultures [27]. In addition to their ability to support neuronal cells in glial-to-neuron communication, EVs can also address the target cells outside the CNS [28,29].

### 2.1. EVs from Oligodendrocytes

**Figure 2:**
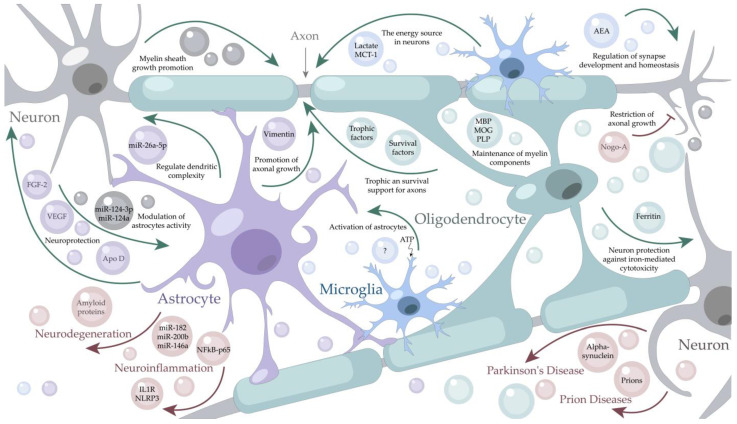
The role of Extracellular Vesicles in the Central Nervous System. Neuron-derived vesicles (grey cells and particles) stimulate myelin sheath growth and modulate the astrocytes’ activity. Oligodendrocyte-derived vesicles (light blue cells and particles) are involved in myelin maintenance, provide neuroprotection and restrict axonal growth. Astrocyte-derived vesicles (purple cells and particles) regulate dendritic complexity, perform neuroprotection functions, and the promotion of axonal growth. Microglia-derived vesicles (blue cells and particles) are a source of energy for neurons and perform activation of astrocytes. Under stress conditions, astrocytes release vesicles loaded with pro-inflammatory proteins (IL1R, NLRP3, NFkB-p65) and miRNAs (miR-146a, miR-182, miR-200b) which could enhance the neuroinflammatory response. Pathological vesicles, released during prion diseases and neurodegeneration can contain amyloid proteins, alpha-synuclein, or even prions and are designated in light red. AEA—N-arachidonoyl ethanolamine; Apo D—apolipoprotein D; FGF-2—fibroblast growth factor 2; IL1R—interleukin-1 receptor; MBP—myelin basic protein; MCT-1—monocarboxylate transporter 1; miR—micro-RNA; MOG—myelin oligodendrocyte glycoprotein; NFkB-p65—nuclear factor kappa-light-chain-enhancer; NLRP3—nucleotide-binding domain-like receptor protein 3; PLP – myelin proteolipid protein; VEGF—vascular endothelial growth factors.

There are multiple sources of EVs in the CNS since all types of neural cells secrete them (Figure 2). One of the CNS EVs sources is oligodendrocytes [30], the myelin-forming cells. Myelin sheath plays an important role in nerve signal transmission protecting nerves and increasing the conduction speed by an order of magnitude [31]. EVs produced by myelinating cells via several delivery mechanisms help to perform the following functions in the nervous system: storage of myelin components, transport of trophic and survival factors into axons, and maintenance of myelin sheath [32]. The exosomes secreted by oligodendrocytes contain myelin-specific lipids and all major myelin components, which constitute up to 70% of the total myelin protein: myelin basic protein (MBP), proteolipid protein (PLP), and myelin oligodendrocyte glycoprotein (MOG) [32]. Oligodendrocyte-derived EVs possess neuroprotective properties providing metabolic support for neurons [33]. These EVs contribute to axonal homeostasis being critical factors for their long-term maintenance and are involved in the regulation of synaptic release [34]. The recently discovered oligodendrocyte-derived EVs loaded with the Ferritin heavy chain have been shown to protect neurons from iron-mediated cytotoxicity [35].

### 2.2. EVs from Neurons

Neurons serve as another source of vesicles in the CNS [36]. Meanwhile, most vesicles produced by neurons are SVs with a diameter of ~40 nm, delivering classical neurotransmitters (glutamate, acetylcholine, GABA, and glycine) and mainly activating the ion channels in the postsynaptic terminals. Another type of neuron-produced EVs—are the so-called large dense-core vesicles (LDCVs) with a diameter in the range of 100–500 nm amounting to only 1~2% when compared to SVs in the CNS [37,38]. They contain a variety of RNAs, proteins, and peptides, including neuropeptides and hormones, which activate G protein-coupled receptors and are involved in the modulation of synaptic activity [39]. LDCVs also store most monoamine transmitters (serotonin, adrenaline, and dopamine) [37], which are responsible for the control of physiological and behavioral functions. It was shown, that EVs from the primary neurons contain Ephs and ephrins, capable of triggering neuronal growth cone collapse [40]. In turn, in addition to synaptic neural communication SVs released by neurons in the CNS help to control synaptic plasticity and promote myelin sheath growth as well as stability in neuron-glial communications [41,42]. Blocking the release of neuronal SVs by toxins makes the oligodendrocytes form shorter sheaths on axons [41] and reduces the sheath number per oligodendrocyte [42] disrupting adequate signal transmission along the nerves. Neurons can also modulate astrocyte activity releasing the exosomes containing miR-124a [43] or miR-124-3p [22], which regulates the expression of the glutamate transporter-1, ultimately contributing to neurotransmitter homeostasis.

### 2.3. EVs from Astrocytes

Astrocytes are another major type of glial cells in the CNS, which produce EVs that deliver miR-26a-5p in the opposite direction—to neurons, thereby regulating dendritic complexity [44]. In general, EVs secreted by astrocytes are considered to be responsible for most of the observed neuroprotective effects [45]. One of the neuroprotective mechanisms is mediated by the transfer of EVs containing Apolipoprotein D to neurons [46]. Apolipoprotein D controls the level of lipid peroxides formed during aging or pathological conditions [47]. Another vesicular cargo specific for astrocytes is vimentin [45]. This intermediate filament protein was shown to promote axonal growth and ameliorate motor dysfunction in a mice model [48]. Astrocyte-derived EVs of the nervous system can contain angiogenic factors such as fibroblast growth factor-2 (FGF-2) and vascular endothelial growth factor (VEGF) [49] which function as the strongest angiogenesis activators [50] and possess neuroprotective properties [51,52]. Small EVs secreted by astrocytes enhance the dendritic spine and synapse formation by primary cortical neurons via TGF-beta signaling [53]. Under stress conditions, astrocyte cells also release EVs containing HSP70 promoting cell survival, preserving normal synaptic transmission, and reducing neuron loss after a traumatic injury. These EVs can also contain synapsin I, involved in synaptic extracellular signaling, and even functional glutamate transporters (EAAT-1/2) playing a crucial role for nervous homeostasis [54]. Thus, astrocytes represent the secretory cell type of the CNS that modulates synapses and ensures neuroprotection.

### 2.4. EVs from Microglia

Microglia are the resident immune cells of the CNS arising from myeloid cells [55]. These cells not only contribute to CNS development and homeostasis but also support and modulate neuronal activity [56]. It was shown, that under IL-4 and IL-13 stimulation, microglia express anti-inflammatory cytokines and factors stimulating tissue repair and extracellular matrix reconstitution [57]. However, microglia also contribute to neuroinflammatory processes [58]; for example, activated microglia release inflammatory factors (IL-1beta, IL-6, TNF-alpha), promoting neuroinflammation [59]. Thus, microglial cells produce a wide range of molecules, including pro- and anti-inflammatory cytokines and interferons [60]. The protein profile of microglial EVs was found to be similar to the protein content of EVs secreted from the antigen-presenting cells—B cells and dendritic cells [61]. The shared proteins embraced MHC (major histocompatibility complex) class II molecules, MHC II-associated chaperone, and cathepsin S. Furthermore, the microglia-derived exosomes contained lactate which serves as an energy source for neurons, expressing isoform 1 of lactate dehydrogenase. These vesicles also contain specific microglial markers-aminopeptidase CD13 and the lactate transporter MCT-1 [61]. Microglia-derived vesicles can regulate synapse development and homeostasis through the delivery of N-arachidonoyl ethanolamine to the surface. This molecule suppresses spontaneous inhibitory transmission in gamma-aminobutyric acid (GABA) neurons via stimulating type-1 cannabinoid receptors [62]. Microglial cells, activated upon TRPV1 stimulation, start shedding microvesicles which promote the metabolism of sphingosine in neurons and elevate the probability of presynaptic release [63]. A more typical stimulus that triggers EV production by microglia is adenosine triphosphate (ATP) mediated by activation of the purinergic receptor P2 × 2R [64]. Meanwhile, the EVs released upon ATP stimulation differ from the constitutively produced EVs and contain proteins implicated in cell adhesion/extracellular matrix organization, autophagy-lysosomal pathway, and cellular metabolism [65]. Such vesicles have a stronger impact on recipient astrocytes than the constitutive EVs. ATP-stimulated EVs dramatically upregulate the production of IL-1beta, IL-6, and TNF-alpha by recipient astrocytes [65].

### 2.5. EVs from Schwann Cells

In the PNS, EVs contribute to axonal growth and regeneration [66]. Peripheral glial cells called Schwann cells regulate axon organization at the molecular level and supply axons with protein synthesis machinery and growth factors via EVs [67]. The exosomes released by Schwann cells were shown to promote the activity of neurons [68]. Schwann-cell-derived exosomes were characterized on the morphological and proteomic level only a few years ago [69]. Currently, the detailed mechanisms of the EV-mediated regulation are not yet fully elucidated; however, proteomic analysis revealed several proteins related to axon regeneration (carboxypeptidase E (CPE), fatty acid-binding protein (FABP5), fibronectin, flotillin-2, major vault protein (MVP), monocarboxylate transporter 1 (MCT1), neuropilin-2 (NRP2), septin-7 (SEPT7), protein disulfide-isomerase A3 (PDIA3), and syntenin-1), which might explain a higher regeneration level in the PNS after injury [69].

### 2.6. Pathogenic EVs

The role of EVs in the nervous system is not confined to a palette of beneficial effects. Exosomes were shown to deliver conventional proteins associated with common neurodegenerative diseases as well as abnormally folded proteins, inducing such a rare and ultimately fatal pathology as prion diseases [66]. Prion disease development caused by microscopic infectious agents is mediated by a fine-tuned system of intercellular interaction. The system efficiently operating under normal conditions can spread the abnormally folded proteins, contributing to clumping the other normal forms of prions in the brain and thus the disease development. Although the role of EVs in the active spreading of prions is still unknown, the presence of prions in the vesicles indicates the importance of thorough research on CNS intercellular communications.

Manifold normally folded proteins, involved in the development of various diseases, are transmitted by EVs as well. Along with EVs that provide trophic support to neuronal cells in glial-to-neuron communication, glial cells can also secrete pathogenic vesicles [28]. Microglia-originated EVs were found to exert pathogenic actions during the development of Alzheimer’s Disease. EVs from patients with Alzheimer’s disease were shown to contain tau protein and to be enriched with innate immune response proteins, neuron-specific proteins, and regulators of amyloid precursor protein (APP) metabolism [70]. Another pathogenic cargo of microglia-derived EVs is α-synuclein, involved in the development of Parkinson’s disease. Studying BV2 cells (a type of microglial cell derived from C57/BL6 murine) provides solid evidence that these cells internalize the pathological exosomal α-synuclein and secrete them inversely owing to exosomes [71]; therefore, promoting the accumulation and transmission of α-synuclein. Upon traumatic brain injury, the levels of microglial EVs increase, and these particles are sufficient to initiate the progressive neuroinflammatory response [72].

Oligodendrocytes have been shown to produce Nogo-A and release the 24 kDa fragment of this axonal growth inhibitor via EVs [73]. The membrane protein Nogo-A is an important factor restricting axonal regeneration and plasticity [74], and its depletion improves vascular repair [75].

Certain EVs produced by astrocyte vesicles play a pathologic role in neuroinflammation. For example, TLR4-mediated astrocyte-derived EVs contain a variety of pro-inflammatory proteins (IL1R, NLRP3, NFkB-p65) and miRNAs (miR-146a, miR-182, miR-200b), which could enhance the neuroinflammatory response [76]. Of note, the negative implications of certain neuroinflammatory processes may be reconsidered, as functional CNS recovery and regular communication between the brain and immune system are maintained by a subtle equilibrium of inflammatory and repair processes [77].

## 3. EVs in Autoimmune Disorders of the Nervous System

Autoimmune disorders can affect the PNS as well as the CNS. The most common CNS autoimmune disorders include multiple sclerosis (MS), acute disseminated encephalomyelitis (ADEM), neuromyelitis optica spectrum disorder (NMOSD), autoimmune encephalitis, etc. In the PNS, autoimmune aggression may affect the peripheral nerves and neuromuscular junction leading to immune-mediated polyneuropathies. Immune-mediated polyneuropathies represent a highly heterogeneous group of diseases and include both acute forms (Guillain-Barre syndrome (GBS), classified into several demyelinating and axonal subtypes) and chronic disorders (multifocal motor neuropathy (MMN), chronic inflammatory demyelinating polyradiculoneuropathy (CIDP)) [78]. These PNS autoimmune disorders are typically treated by plasma exchange, administration of intravenous immunoglobulin, and corticosteroids [79]. Peripheral nerve injuries lead to sensory and motor dysfunction in the respective parts of the body, but in contrast to the CNS, the PNS has a high regenerative capacity [80]. As many autoimmune neurological disorders are quite rare, the exact role of EVs in their development is still to be pinpointed. Currently, there are no known approved sensitive EVs-based biomarkers or characteristics of EV available for monitoring the onset, progression, or therapeutic response for the majority of autoimmune pathologies. Below, we confine our attention only to the autoimmune neurological pathologies that are evidenced to involve EVs.

### 3.1. EVs in MS Diagnostics

MS is an inflammatory autoimmune demyelinating disorder of the CNS. It mostly strikes young and middle-aged people and leads to a gradual disability. MS is a highly heterogeneous disease with unknown etiology; it is manifested with characteristic white matter lesions, breakdown of the BBB, loss of oligodendrocytes, and degeneration of axons and neurons [81]. MS progression is believed to arise from malfunction both of B- and T-cell compartments and is associated with auto-reactive antibodies [82,83,84], auto-aggressive B cells [85,86], auto-aggressive T cells [87], and predominance of the certain MHC II alleles [88]. MS can only be diagnosed after the first manifestation, often years later after its original onset [89]. Therefore, the development of novel diagnostic markers is of great interest and importance, since early treatment allows for better control and suppression of the disease. To date, there are several approved strategies for MS treatment, but all of them aim at reducing clinical symptoms and do not lead to a complete recovery.

Recent studies imply that in the future, employing EVs might resolve some of the limitations of MS treatment and diagnosis. EVs in CSF of MS patients were first observed by Scolding et al. in 1989 [90]. Later an increase in the amount of EVs was found both in CSF and in the serum of MS patients compared with the healthy donors [91]. It was shown, that EVs from CSF or serum of MS patients may be present upon neuroinflammation, BBB damage, and other pathophysiological reactions [91]. For example, circulating neuron-originated and astrocyte-originated EVs from the serum of MS patients indicate synaptic loss [92]. There was a statistically significant difference in the levels of synaptopodin (post-synaptic protein) and synaptophysin (pre-synaptic protein) from the neuronal EVs between MS patients and healthy donors. Meanwhile, astrocyte-derived EVs from MS patients contained higher levels of the complement components (C1q, C3, C3b/iC3b, C5, C5a, and Factor H) as compared with control samples [92]. EVs can also be used to evaluate the MS clinical status of the patient since elevated CD31+ EVs were associated with the relapse of the disease [93]. Another useful source of information is miRNA profiling, as they are involved both in immune regulation and myelination. As EVs are enriched with miRNAs and ensure their stability compared to the blood serum, EV-derived miRNAs can serve as potential biomarkers for various diseases [94]. In MS patients, miRNAs from EVs were shown to provide information on the status of the CNS. Cuomo-Haymour and colleagues showed the association of the miR-451a and miR-25-3p up-regulation in EVs with the RRMS course [95].

**Figure 3:**
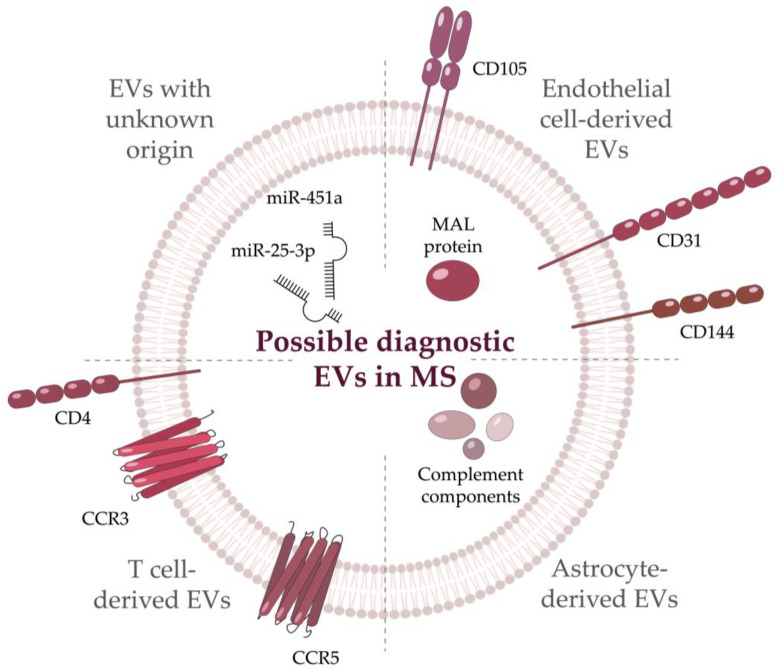
Potential EVs’ biomarkers emerging during Multiple Sclerosis. Biomolecules grouped according to their origin. CCR—C-C Motif Chemokine Receptor; MAL—Myelin And Lymphocyte protein; miR—micro RNA.

The BBB damage occurs at an early stage of MS development. It is associated with the destruction of endothelial cells and the development of the pro-inflammatory microenvironment. Thus, enhanced EVs release from the endothelial cells is associated with their stimulation or injury, such EVs could serve as a biomarker of the BBB damage during MS [96]. The CNS-endothelial EVs contain a combination of pan-endothelial markers (CD31, CD105, or CD144) with myelin and lymphocyte protein (MAL). The population of specific CNS-endothelial EVs is considered a biomarker of BBB permeability and the acute phase of disease in patients with the active form compared to healthy donors and stable MS patients [96]. Applying CD31-positive EVs as a potential biomarker for MS is limited by their partial specificity as they are also produced in type 2 diabetes mellitus [97] and cardiovascular diseases as well [96]. Platelet-derived EVs are also increased in the blood serum of MS patients [98] and may serve as MS diagnostic markers after further research. Of note, the number of certain vesicles may decline during disease progression. For example, a reduced level of CD19+/CD200+ (the marker of immature B cells) EVs from CSF was observed in MS patients. The authors also linked an increase in the CCR3+/CCR5+ (a subset of CD8 memory T cells), CD4+/CCR3+ (Th2 cells), and CD4+/CCR5+ (Th1 cells) EVs from CSF with the presence of lesions in the brain and spinal cord [99]. Current candidates for future EV biomarkers of MS are summarized in Figure 3. Further describing EVs in CSF might shed more light on intercellular interactions in the CNS.

### 3.2. EVs in NMOSD Diagnostics

NMOSD, also known as the Devic syndrome, is a chronic inflammatory autoimmune disease, characterized by severe attacks of acute optic neuritis and transverse myelitis [100]. The first manifestation of this pathology is autoimmune astrocytopathy followed by the secondary damage of oligodendrocytes and neurons [101]. Non-treated NMOSD can lead to visual and motor dysfunction [102]. In 80% of cases, NMOSD is caused by autoreactive autoantibodies to Aquaporin-4 [102,103], the most abundant water channel in the CNS [104], but in some cases, the antibodies against MOG have also been suggested to induce NMOSD [105].

There are quite a few animal models of NMOSD [106], however, the existence of a representative model is controversial. Correct and prompt diagnosis of NMOSD is very important because acute and severe damage of the optic nerve ultimately results in complete vision loss. EVs might help to detect tissue and cell damage in the CNS during NMOSD. Exosomal proteome analysis of the samples from MS and NMOSD patients revealed a panel of NMOSD-specific proteins comprising GFAP, C4b-binding protein, and haptoglobin-related protein. Two of these proteins (C4b-binding protein, haptoglobin-related protein) were present in EVs-enriched fraction, but not in total CSF [107].

EVs containing Aquaporin-4 might serve as more specific markers of NMOSD. Aquaporin-4 positive EVs can induce widespread neuroinflammation and production of autoantibodies. An increased amount of EVs with Aquaporin-4 in CSF may be used for early diagnosis of NMOSD [108]. Another study identified specific exosomal miRNAs pattern in NMOSD patients with absence in healthy donors. Notably, some of them (hsa-miR-122-3p and hsa-miR-200a-5p) were upregulated in acute NMOSD, while remaining unchanged during NMOSD remission [109].

### 3.3. EVs in Diagnostics of Autoimmune Encephalitis

Autoimmune encephalitis is a non-infectious form of brain inflammation characterized by the development of auto-aggressive antibodies against the neuronal cell surface or synaptic proteins [110]. Earlier, infectious encephalitis was considered to be the most common type of encephalitis that impeded the diagnostic criteria for autoimmune encephalitis to be developed [110]. Yet, the study by Dubey et al. showed that the autoimmune form of encephalitis occurred at the same rate as the infectious one [111]. The most frequently identified antigen targets in this disease are MOG and GAD65 (glutamic acid decarboxylase-65), while certain types are caused by the autoantibodies against other antigens like gamma-aminobutyric acid-B receptor (GABA_B_R), Aquaporin-4, glial fibrillary acidic protein, leucine-rich glioma-inactivated-protein-1, collapsin response-mediator protein-5, N-methyl-D-aspartate receptor (NMDAR), contactin-associated protein-like 2 (CASPR2) and glial fibrillary acidic protein-α [111]. Autoaggression leads to localized inflammation in various CNS structures, resulting in significant variability of clinical features including acute or subacute epileptic seizures, cognitive impairment, and mental symptoms [112]. The inflammation in autoimmune encephalitis mainly affects the limbic system, but extensive changes in the temporal cortex, basal ganglia, brain stem, frontal and parietal cortex are also not unusual and may be involved in the inflammation based on the unique antibodies pattern in each case [113]. Autoimmune encephalitis is considered to be a paraneoplastic disease, that is, it is commonly accompanied by the presence of an occult tumor that can stimulate autoantibody production.

It is well established that CSF in patients with autoimmune encephalitis is characterized by elevated lymphocyte and protein levels, including the presence of oligoclonal bands [114]. Wherein, only at the end of 2020, EVs bearing neuronal autoantigen aggregates were found to be enriched during autoimmune encephalitis [115]. CSF and the serum of patients with autoimmune encephalitis contained the antigens targeted by the antibodies developed in a corresponding patient. The exosomes containing NMDAR, GABA_B_R1, LGI1, CASPR2, or GluR1/2 subunits of AMPAR were detected in the patient samples. Moreover, the antibodies against the mentioned antigens were present in the sera of C57BL/6 J mice, immunized with exosomes isolated from antibody-positive autoimmune encephalitis patients. Later the same authors identified a panel of ten miRNAs (especially miR-301a-5p, miR21-5p, miR-128-5p, miR-155-5p, miR-34a-5p, miR-326-5p, miR132-5p, miR-29b-5p) with enhanced and eight miRNAs (especially miR-20a-5p) with reduced expression level during autoimmune encephalitis [116]. It should be noted that despite the evidence of EVs appearing during autoimmune encephalitis, Herpes Simplex Encephalitis evokes significantly higher EVs levels in CSF. Therefore, the contribution of EVs to the manifestation of clinical symptoms in autoimmune encephalitis has yet to be elucidated.

### 3.4. EVs in GBS Diagnostics

GBS is an immune-mediated inflammatory disease of the PNS, which is supposedly triggered by bacterial or viral infections [117,118]. It is a rare, but potentially fatal disorder, especially in the absence of timely treatment; GBS occurs predominantly in males (Leonhard et al., 2019). It is characterized by rapidly progressing, symmetrical weakness in legs and arms, resulting in acute areflexic paralysis [119]. Disease development is accompanied by massive lymphocytic infiltration and degradation of the myelin sheath of the peripheral nerves. GBS is a highly heterogeneous disease with several clinical outcomes. The most frequent subtypes are acute inflammatory demyelinating polyneuropathy (AIDP) and acute motor axonal neuropathy (AMAN) [120]. The other rarer subtypes of GBS are acute motor and sensory axonal neuropathy (AMSAN) and Miller Fisher syndrome (MFS) observed only in about 1–5% of GBS patients in the Western populations and up to 25% in Asians [121]. Autoantibodies against components of peripheral nerves (gangliosides) are strongly associated with AMAN and Miller–Fisher syndrome, but not with AIDP [122]. The localization of these four ganglioside autoantigens—GM1, GD1a, GT1a, and GQ1b—has been associated with distinct clinical patterns of GBS [122].

Although the GBS etiology is poorly understood, the role of CD4+ T cells and macrophages in disease initiation and development is well documented [123]. The exosomes derived from M1 and M2 macrophages have opposing impacts on the development of experimental autoimmune neuritis (EAN), a well-accepted animal model of GBS mimicking the AIDP subtype [124]. The exosomes released by M1 macrophages were shown to promote EAN development and can directly stimulate IFN-γ expression in the Th1 effector cells. Wherein, the exosomes from M2 macrophages were able to attenuate EAN, although unable to completely suppress Th1 response.

### 3.5. EVs in Amyotrophic Lateral Sclerosis Diagnostics

Amyotrophic lateral sclerosis (ALS), also called Lou Gehrig’s disease, is a fatal neurodegenerative disorder characterized by progressive muscle paralysis reflecting the death of both upper and lower motor neurons in the primary motor cortex, corticospinal tract, brainstem, and spinal cord [125]. The disease, which manifests itself commonly in middle age, has an incidence of 2.31–2.35 per 100,000 persons per year in Europe and North America [126]. Typically, patients die of respiratory failure after 2–5 years from the onset due to the lack of effective treatment. The causes of ALS and the actual mechanisms of neuronal death are currently unknown. Environmental factors, older age, male gender, and a family history of ALS have been proposed as the main risk factors. ALS cannot be unequivocally called an autoimmune disease, however, the evidence of the contribution of autoimmune mechanisms to its development [127,128] compelled us to review this disease in more detail. ALS is characterized by the infiltration of T-lymphocytes and macrophages in the CNS [129,130,131], immunoglobulins from ALS patients cause the degeneration of mice motor neurons [132,133,134]; the role of the autoimmune inflammation in ALS is further supported by the increased CSF and serum levels of IL-17 and IL-23 produced predominantly by Th17 [135].

Remarkable research efforts have been recently directed toward studying the differential gene expression as well as the changes in non-coding RNA production by neuronal and glial cells during normal brain development and pathological states. Unfortunately, to date, there are no effective tests for ALS diagnosis and classification. Therefore, using CNS-derived EVs as novel biomarkers for ALS diagnosis is highly feasible. The microvesicles derived from the plasma of ALS patients were enriched in pathological proteins SOD1, TDP-43, and FUS compared to healthy donors [136]. Herewith, the mean size of microvesicles and exosomes was increased in ALS patients. Moreover, EV-encapsulated TDP-43 was shown to stimulate monocytic activation [137] and exert higher toxicity than free TDP-43 [138]. On the contrary, the inhibition of exosome secretion by the inactivation of neutral sphingomyelinase 2 with GW4869, provoked TDP-43 aggregation in Neuro2a cells and exacerbated the disease phenotypes of human TDP-43A315T transgenic mice [139]. Notably, the dipeptide repeat proteins (DRPs) capable to form aggregates in the CNS of ALS patients, can spread between cells via exosomes [140]. With regard to non-coding RNA, about 20 differentially expressed miRNAs in EVs have been identified in ALS patients [141], among which reduced miR-27a-3p [142] and miR-9-5p [143] levels in serum exosomes can be specifically emphasized.

### 3.6. Therapy of Experimental Autoimmune Encephalomyelitis

Apart from various diagnostic applications, EVs are endowed with enormous therapeutic potential (Table 1). Considering the novelty of EV-based drug development, currently, only preclinical studies are being conducted. Experimental autoimmune encephalomyelitis (EAE) is the most used versatile experimental animal model of MS [144], which allowed the development of several first-line disease-modifying therapies approved by the FDA [145]. One of the promising areas of experimental MS therapy is harnessing the vesicles derived from mesenchymal stem cells (MSCs). Cell-free MSC-based therapy is safer than MSC transplantation but retains all the benefits of MSCs [146]. As MSCs can secrete a large number of EVs acting in a paracrine manner [147], this therapy can be a novel promising approach with high potential to treat various diseases [148,149]. Studying the function of EVs derived from the IFN-gamma-activated MSC on the EAE model showed the potential of these EVs to suppress PBMC proliferation, reduce the expression of pro-inflammatory cytokines, and enhance regulatory T cell induction [150]. These EVs were demonstrated to reduce neuroinflammation and demyelination in EAE mice. EVs from bone marrow MSCs exerted neuroprotective immunoregulatory functions and promoted the polarization of microglia from M1 toward M2 phenotypes in the brain and spinal cord of the rats with EAE. Treatment with MSCs-derived EVs stimulated IL-10 and TGF-beta secretion and reduced the TNF-alpha production in the rats [151]. Bone marrow MSC exosomes also elevated the number of oligodendrocytes and promoted remyelination in EAE models [152]. Guinti et al. [153] demonstrated the impact of miRNA delivered by MSC-derived EVs. Eight miRNAs (miR-467f, miR-466q, miR-466m-5p, miR-466i-3p, miR-466i-5p, miR-467g, miR-3082-5p, and miR-669c-3p) targeted to genes encoding inflammatory molecules were found to reduce the expression of neuroinflammation markers in EAE-induced mice after administration of MSC-derived EVs. Another source of stem-derived EVs is the placenta. Unlike bone marrow-derived MSCs, placenta-derived MSCs express human leukocyte antigen-G molecules on their surface, which may be useful in inhibiting the cytotoxicity of Natural Killer cells and CD8+ T cells [154]. The *in vitro* study demonstrated the promoted maturation of oligodendrocyte precursor cells treated with EVs from placenta-derived MSCs, and further, the *in vivo* study showed a decrease in myelin loss in the spinal cord of EAE mice [155]. EVs from adipose-derived MSCs can be used in MS therapy as well. The intravenous administration of these EVs attenuated EAE in mice by reducing the proliferative activity of T cells, leukocyte infiltration, and demyelination [156]. Another study showed the preventive effects of the administration of such EVs to MOG_35-55_-immunized mice before the manifestation of the EAE clinical signs. Mice treated with adipose-derived MCS EVs prior to the disease onset showed a reduced clinical score as well as reduced demyelinating areas and a declined number of CD3+ infiltrating T cells in the CNS, according to the neuropathological studies [157].

Stem cells are not the only source of therapeutic EVs. Oligodendrocyte-derived EVs have been reported as effective antigen-specific therapeutics. On the one hand, EVs preparations (both exosomes and microvesicles) containing major MS autoantigens (PLP, MOG, MBP) suppressed neuroinflammation in an antigen-dependent manner. In this case, immune tolerance was achieved by inducing the immunosuppressive monocytes and apoptosis of the autoreactive CD4+ T cells [158]. On the other hand, EVs with fibrinogen isolated from human blood were found to cause a unique spontaneous relapsing-remitting disease phenotype and deteriorate the EAE pattern in MOG_35-55_ immunized mice. These EVs induced CD8+ T cells which are not common in the EAE model [159]. Shen et al. [160] demonstrated the ability of neutrophil-derived nanovesicles to affect white matter loss and modulate neuroinflammation in mice with EAE. These effects are mediated by the scavenging of myelin debris by microglia, induced by upregulated expression of nuclear factor E2-related factor 2 (NRF2).

Engineered EVs could be promising tools for MS treatment as well. Casella and co-authors designed the microglial cell line, releasing vesicles targeting phagocytes and containing the anti-inflammatory cytokine IL-4 with multifunctional glycoprotein Lactadherin on its surface [161]. A single injection of these EVs into the brain (cisterna magna) mitigated neuroinflammation and alleviated clinical signs in the EAE mice. The group of Xing Li utilized modified neural stem cells with highly expressed ligand PDGF-A as a source of EVs (EVPs) for targeted delivery to oligodendrocytes [162]. Moreover, they demonstrated that analyzed EVPs were able to embed triiodothyronine, a thyroid hormone that is critical for oligodendrocyte development but induces serious side effects upon systemic and excessive administration. Injecting the EVPs loaded with low dosages of triiodothyronine significantly reduced EAE symptoms, enhanced oligodendrocyte survival, suppressed myelin damage, and promoted myelin regeneration. Later in 2022, the same group used EVPs as a targeted carrier for encapsulating Bryostatin-1, a natural compound with anti-inflammation properties [163]. Its administration also significantly ameliorated EAE, reduced the infiltration of pro-inflammatory cells, protected BBB integrity, and abrogated myelin loss and astrogliosis. Although the authors did not compare the efficiency of EVPs loaded with triiodothyronine or Bryostatin-1, they showed the ability of stem cells-derived engineered EVs for highly specific drug delivery to oligodendrocytes, providing powerful therapeutic effects for the EAE treatment. Another study aimed at designing EVs with therapeutic proteins exposed on the membrane surface [164]. Different EV-loading moieties were screened to obtain EVs with cytokine-binding domains derived from the tumor necrosis factor receptor 1 (TNFR1) and IL-6 signal transducer (IL-6ST) that lack their signaling domains. These domains inhibited TNF-alpha and the IL-6/sIL-6R complexes acting as decoys for these pro-inflammatory cytokines (TNF-alpha and IL-6, respectively). The engineered EVs decreased cytokine levels in the spinal cord of EAE mice and reduced the clinical score [164]. Utilizing engineered EVs can elevate the loading limit of the vesicles, improve delivery specificity, and minimize possible off-target molecules. In recent work, engineered EVs were shown to deliver proteins specifically to the antigen-presenting cells [165] and facilitate the loading of the target protein [166]. Such EVs can prove a promising tool for delivering protein cargo to the immune cells and thus provide solid ground for future MS therapeutics.

## 4. Conclusions

EVs are promising molecular tools, which have demonstrated excellent results in a range of animal studies as biomarkers, therapeutic agents, and even efficient drug delivery carriers [167]. They have been actively studied in cancer [168], cardiology [169], aging [170], acute injuries [171], and infectious diseases [172].

Although there are significant advances in the use of EVs, utilizing EVs in diagnosis and therapy still has certain limitations arising from the knowledge gaps in their composition. Studying EV proteome and possible off-target molecules is essential as they may have yet unpredictable side effects [159].

Unfortunately, emerging approaches employing EVs as potential biomarkers for autoimmune diseases are limited by the exceptional heterogeneity of EV subpopulations and the complicated purification process of EVs from body fluids. Quite a few studies showed their promising diagnostic potential, however, only a few of them made it to the clinic. The number of studies devoted to EVs is growing, but some of them employ different methods of purification, which makes it difficult to compare them with each other and this calls for a systematic review. Owing to that it is important to report and standardize all protocol details applied in EVs studies. It is also important to provide full information about the specifics of biofluid collection or EVs-releasing cell culture and harvesting conditions according to minimal information for studies of extracellular vesicle guidelines approved by the international society for extracellular vesicles [3]. To date, it is well known that vesicles are involved in the onset and progression of various diseases, but so far, the list of potential EV markers is relatively short and can be applied to a limited range of diseases. It is important to bear in mind that quite a few publications, analyzing EVs in the serum or CSF of patients utilize ultracentrifugation for EV purification, therefore, it is not clear whether the identified components represented the ultimately vesicular components or were just co-isolated from free serum or CSF. Thus, further use of the vesicles in clinical practice requires more thorough research of their biogenesis, protein and RNA composition, purity, the content of non-target molecules, and their possible influence on the cells.

## Figures and Tables

**Table 1:** Efficacy of EVs-based therapy of EAE.

Ref.	EVs Source	EVs Isolation	Animal Model of Demyelination	EVs Administration	Main Outcomes
[148]	Human bone-marrow-derived MSCs activated with 10 ng/mL of IFNγ	(1) Culture media were centrifuged at 300× *g* for 10 min. ↓ (S) (2) 16,000× *g* for 20 min. ↓ (S) (3) 120,000× *g* for 2.5 h at 4 °C. ↓ (S) (4) Pellet was reconstituted in PBS	EAE induction with MOG_35-55_ in 6–8 weeks old female C57BL/6J mice.	150 μg (1.06 × 10^9^ ± 9.6 × 10^7^ particles per NTA) of EVs were injected intravenously (i.v.) at the peak of the EAE (15–20 days).	(1) The suppression of PBMC cell proliferation, reduction of proinflammatory cytokines and enhanced induction of Tregs in vitro. (2) The reduction of neuroinflammation and demyelination and improvement in functional outcomes in chronic EAE.
[149]	Rat bone-marrow-derived MSCs	(1) Culture media were centrifuged at 300× *g* for 10 min. ↓ (S) (2) 2000× *g* for 20 min. ↓ (S) (3) 10,000× *g* for 30 min. ↓ (S) (4) 100,000× *g* for 70 min-twice.	EAE induction with guinea pig spinal cord homogenate in female Sprague Dawley (SD) rats.	100 μg (low dose) or 400 μg (high dose) of EVs were injected i.v. 24h after EAE induction.	(1) The improvement in neurobehavior score and prevention of weight loss. (2) The reduction of TNF-alpha and the enhancement in IL-10 and TGF-beta secretion. (3) Polarization of microglia from an M1 phenotype to an M2 phenotype. (4) High doses of EVs attenuated the infiltration of inflammatory cells and demyelination in spinal cords of EAE rats.
[150]	Monkey bone-marrow-derived MSCs	(1) Culture media were centrifuged at 250× *g* for 5 min. ↓ (S) (2) 3000× *g* for 30 min. ↓ (S) (3) 0.22 μm filtration. ↓ (F) (4) 100,000× *g* for 2 h at 4 °C (5) Pellet was reconstituted in PBS	(1) EAE induction with MOG_35-55_ in 6–8 weeks old female C57BL/6 mice. (2) White matter toxicity demyelination model induced by CPZ in male 8 weeks old C57BL/6 J mice	(1) 5 × 10^10^ of EVs were injected i.v. twice a week initiated on day 10 post EAE induction for 4 weeks. (2) 5 × 10^10^ of EVs were injected i.v. once a week initiated on the day of the CPZ diet withdrawal for 2 weeks	(1) The improvement in neurological and cognitive functional recovery. (2) The increased myelination and new generation of oligodendrocytes in the spinal cord of EAE mice. (3) The decrease of neuroinflammation and polarization of microglia from M1 to M2 phenotype. (4) Inhibited the Toll-like receptor 2 (TLR2)/interleukin-1 receptor-associated kinase 1 (IRAK1)/NF-κB signaling pathway in spinal cord tissues.
[151]	Murine bone-marrow-derived MSCs activated with IFNγ and stimulated with 1 mM ATP	(1) Culture media were centrifuged at 2000× *g* for 20 min at 4 °C. ↓ (S) (2) O/n incubation with 0.5 volume of Total Exosome Isolation Kit (Invitrogen) at 4 °C. (3) 10,000× *g* of o/n incubated sample for 1 h at 4 °C. (4) Pellet was reconstituted in PBS.	EAE induction with MOG_35-55_ in 6–8 weeks old female C57BL/6J mice.	EVs (yield from 3–10 × 10^6^) were injected i.v., on alternate days for 8 days or intraperitoneally daily for 6 days from the onset of clinical symptoms.	(1) The downregulation of pro-inflammatory markers (TNF, IL-1beta, IL-6, and Nos2) in spinal cord tissue. (2) No effect on disease course, independently of the administration route.
[153]	Human placenta-derived MSCs cultured with FGF and EGF	(1) Culture media were centrifuged at 4 °C at 300× *g* for 10 min. ↓ (S) (2) 2000× *g* for 30 min. ↓ (S) (3) 0.22 μm filtration. ↓ (F) (4) Concentration through Amicon Centrifugal Filter Units with a 100 kDa MW cutoff (Millipore Sigma) (5) 8836× *g* ↓ (S) (6) 112,700× *g* for 90 min. (7) Pellet was reconstituted in PBS.	EAE induction with MOG_35-55_ in 3-month-old female and male C57BL/6J mice.	1 × 10^7^ (low dose) or 1 × 10^10^ (high dose) of EVs were injected i.v., on day 19 post EAE induction.	(1) The improvement in motor functions. (2) The reduction of oligodendrocyte damage in spinal cords of EAE mice. (3) The decrease in myelin loss.
[154]	Human adipose-derived MSC	(1) Culture media were centrifuged at 4 °C at 500× *g* for 20 min. ↓ (S) (2) 18,000× *g* for 30 min. ↓ (S) (3) 0.22 μm filtration. ↓ (F) (4) 120,000× *g* for 90 min. (5) Pellet was reconstituted in PBS.	EAE induction with MOG_35-55_ in 6–8 weeks old female C57BL/6 mice.	60 μg of EVs were injected i.v. on day 10 postimmunization.	(1) The decrease of maximum mean clinical score in EV-treated mice. (2) Reduced splenocyte proliferation (3) The significant reduction in the demyelination areas and inflammatory infiltrate cells.
[155]	Murine adipose-derived MSC cultured with HB-EGF	(1) Culture media were centrifuged at 4 °C at 80× *g* for 5 min. ↓ (S) (2) 1300× *g* for 10 min. ↓ (S) (3) 0.22 μm filtration. ↓ (F) (4) Concentration through membrane concentrator (MWCO 5K, Corning Spin-X) at 3200× *g* for 90 min at 4 °C. (5) 100,000× *g* for 60 min at 4 °C-twice.	EAE induction with MOG_35-55_ in 6–8 weeks old female C57BL/6 mice.	5 μg of nanovesicles were injected i.v. on days 3, 8, and 13 postimmunization (preventive protocol) or were injected i.v. on days 12, 16, and 20 postimmunization (therapeutic protocol)	(1) Amelioration of clinical score in EAE mice then utilizing preventive protocol of EVs injection. (2) The reduction of spinal cord inflammation and demyelination (preventive protocol). (3) No change in the clinical course of EAE when injected after the disease onset.
[158]	Mouse neutrophil cell line	(1) Serial extrusion (1000 nm, 400nm, 200 nm pore sizes) of the cell suspension. (2) Obtained nanovesicles were subjected to density gradient (10% to 50% OptiPrep) ultracentrifugation at 100,000× *g* for 2 h at 4 °C.	EAE induction with MOG_35-55_ in 6–8 weeks old female C57BL/6 mice.	50 μg of nanovesicles were administered daily from day 3 after immunization.	(1) Modulation of neuroinflammation in mice. (2) Regulation of white matter loss. (3) Gene ontology showed decreased neuroinflammation-related pathways. (4) Promotion of myelin debris clearance of microglia with subsequent cellular inflammation resolution.

CPZ-Cuprizone; EAE-Experimental autoimmune encephalomyelitis; EGF-Epidermal growth factor; FGF-Fibroblast growth factor; HB-Heparin-binding EGF-like growth factor; i.v.-intravenously; IFN-Interferon; IL-Interleukin; MOG-Myelin oligodendrocyte glycoprotein; MSC-Mesenchymal stem cells; o/n-overnight; PBMC-Peripheral blood mononuclear cell; PBS-Phosphate buffered saline; TGF-Tumor growth factor; TNF-Tumor necrosis factor; ↓ (S)-the supernatant from this step was used for further EVs isolation step; ↓ (F)-the filtrate from this step was used for further EVs isolation step.

## Data Availability

Not applicable.
